# Implicit and Explicit Regularization for Optical Flow Estimation

**DOI:** 10.3390/s20143855

**Published:** 2020-07-10

**Authors:** Konstantinos Karageorgos, Anastasios Dimou, Federico Alvarez, Petros Daras

**Affiliations:** 1 The Visual Computing Lab, Information Technologies Institute, Centre for Research and Technology Hellas, 57001 Thessaloniki, Greece; dimou@iti.gr (A.D.); daras@iti.gr (P.D.); 2 Universidad Politécnica de Madrid, 28040 Madrid, Spain; fag@gatv.ssr.upm.es

**Keywords:** optical flow, regularization, semantic segmentation, motion consistency, coordconv

## Abstract

In this paper, two novel and practical regularizing methods are proposed to improve existing neural network architectures for monocular optical flow estimation. The proposed methods aim to alleviate deficiencies of current methods, such as flow leakage across objects and motion consistency within rigid objects, by exploiting contextual information. More specifically, the first regularization method utilizes semantic information during the training process to explicitly regularize the produced optical flow field. The novelty of this method lies in the use of semantic segmentation masks to teach the network to implicitly identify the semantic edges of an object and better reason on the local motion flow. A novel loss function is introduced that takes into account the objects’ boundaries as derived from the semantic segmentation mask to selectively penalize motion inconsistency within an object. The method is architecture agnostic and can be integrated into any neural network without modifying or adding complexity at inference. The second regularization method adds spatial awareness to the input data of the network in order to improve training stability and efficiency. The coordinates of each pixel are used as an additional feature, breaking the invariance properties of the neural network architecture. The additional features are shown to implicitly regularize the optical flow estimation enforcing a consistent flow, while improving both the performance and the convergence time. Finally, the combination of both regularization methods further improves the performance of existing cutting edge architectures in a complementary way, both quantitatively and qualitatively, on popular flow estimation benchmark datasets.

## 1. Introduction

Optical flow estimation is a prerequisite step in a variety of computer vision problems, ranging from obvious ones, such as object tracking [1], action recognition [2], motion analysis [3], and video stabilization [4], to more sophisticated ones, such as monocular depth estimation [5], multi-frame super resolution [6], and 3D object reconstruction in immersive environments [7]. The computation of optical flow is, however, an ill-posed problem in its naive formulation, as there are multiple valid solutions.

Training a Deep Neural Network (DNN) network for a complex problem is generally a cumbersome procedure, highly sensitive to the training set and the parameters used. In the relevant literature, different strategies have been followed to improve accuracy by either modifying the objective function or controlling the biases, also known as regularization methods. Regularization can be defined as any strategy employed to improve the training procedure of a neural network by imposing problem-specific restrictions. It has been shown that regularization can improve the behavior of DNNs [8], mitigating the inevitable bias of the training set and guiding them towards more generalized solutions.

**Figure 1:**
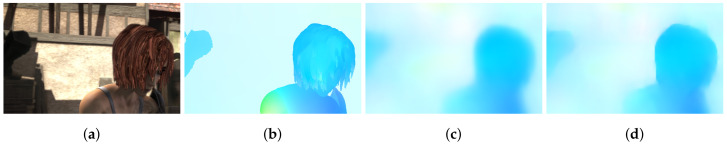
Illustration of global smoothing deficiency. (**a**) Reference image. (**b**) Ground truth optical flow. (**c**) Flow from a globally regularized network. (**d**) Flow from a locally regularized network. Notice that the silhouette in panel (**d**) is visibly less blurry than in panel (**c**).

Motion consistency is a well-established principle used for the regularization of optical flow estimation networks. Neighboring points of the scene are assumed to move uniformly; a hypothesis that is accurate in most cases. However, this assumption fails significantly on object boundaries, as depicted in Figure 1, as well as on joints of non-rigid objects. Consequently, methods that adopt the motion consistency principle tend to suffer from “soft”and inaccurate edges between objects. This phenomenon is more evident when two neighboring objects are moving in opposing directions. Edge maps have already been used in optical flow estimation [9] in order to enable special computational strategies on the edges. However, these methods still rely on low-level edge extraction and often fail to distinguish possible motion-separating edges from simple texture patterns or variations. In this paper, we show that semantically richer information can be used to improve flow estimation accuracy and motion consistency.

Optical flow and semantic segmentation may be seen as disparate modalities given that the former is about motion and the latter is about scene understanding. They are intertwined, though, as we can easily reason about the possible motions of a scene just by looking at its semantic segmentation (e.g., buildings never move) and, on the other hand, understand the semantics of an object just by looking the patters of its motion (e.g., human walking has a distinct pattern). The close relationship of the aforementioned modalities is confirmed in the literature, as they are jointly exploited [10,11]. The affinity of the two research domains is demonstrated in [12] and further grounded in recent neuroscience reports such as in [13,14].

The current work presents two regularization methods: one explicit and one implicit. The first one explicitly penalizes motion irregularities in the resulting flow field, leading to smoother and more accurate results. The second method indirectly affects the resulting flow field and is, thus, referred to as implicit. Its regularizing effects are thoroughly analyzed quantitatively and qualitatively.

The explicit regularization method aims to guide any optical flow estimation network to better model the correlation between motion flow and texture on the edge of an object. The training procedure is regularized by selectively imposing the motion consistency constraint on semantically coherent local regions of the image. For this purpose, a new loss function is introduced that takes into account the semantic segmentation mask of the examined scene to reason on the validity of motion consistency. Based on this approach, the network learns to distinguish inter- and intra-object edges and enforce smoothness accordingly. At the inference stage, the network produces sharper flow with less prevalent motion field leaks across objects and higher motion consistency within each object, without requiring any additional input or modification.

The implicit regularization method aims to improve the training procedure by reducing the legitimate solution space. DNNs are designed to be translation invariant in order to improve their ability to detect textures and objects within an image. This translation invariance stems mainly from the pooling layers of the network, while convolutional layers are also contributing to it. While in some applications this is a desirable property, a recent work [15] explored its potential deficiencies for different types of computer vision applications. In this work, we argue that for optical flow estimation, translation invariance introduces learning perturbations impeding the efficient training of the network. Thus, by removing the translation invariance, the network’s training procedure is implicitly regularized, leading to a faster convergence with increased accuracy.

Moreover, an exploration of both regularization strategies, explicit and implicit, as well as their complementarity, is explored in order to better understand the role of regularization in optical flow estimation. For this purpose, the widely adopted flow estimation network FlowNet2 [16] and its building blocks are used as a baseline. An ablation study is performed to verify the validity of our claims, gradually integrating the regularization strategies and building up in network complexity. The evaluation of the proposed methods against the baseline is performed on popular datasets used as a benchmark for optical flow estimation.

The contributions of this work can be summarized as follows.

- A new loss function for explicit regularization is proposed that takes into account the semantic segmentation mask of the examined scene, enforcing motion consistency within the object and sharpness on the boundaries. Different loss designs are extensively explored and tested at multiple stages of the baseline architecture.
- The use of pixel coordinate information as an implicit regularizer in optical flow estimation networks is proposed. An in depth exploration of the role of translational invariance on the quality of flow estimation is performed to validate our claims.
- The proposed regularization methods, along with their complementarity, have been extensively assessed on the optical flow estimation task in popular publicly available datasets and insights on the training procedure are provided.

## 2. Related Work

**Figure 2:**
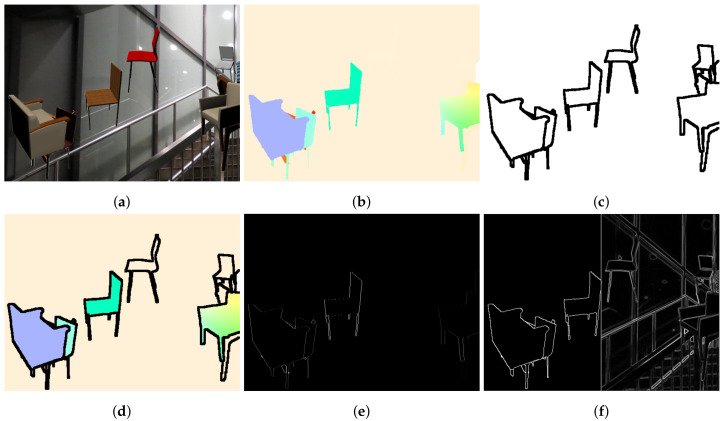
(**a**) Sample from FlyingChairs dataset. (**b**) Ground truth optical flow. (**c**) Corresponding $M_{sem}$ mask. (**d**) Overlay of $M_{sem}$ and GT flow. Notice how enclosed segments have a smooth flow. (**e**) Motion edges. Notice that it is concentrated on boundaries. (**f**) Comparison of semantic edges with a typical edge mask. Notice how they better correlate to motion edges.

Regularization of optical flow estimation methods goes back to Horn and Schunck [17]. They formulated optical flow estimation as a global energy minimization task, which is intractable without regularization in the form of global motion consistency. Such variational methods form an objective function comprised of a data and regularization terms: $L=E_{data}+E_{reg}$. Plethora of work exists on improving the effects of $E_{reg}$. To avoid the oversmoothing effect of total variation regularization, Shulman et al. [18] utilized a nonlinear gradient weighting term. Nagel and Enkelmann [19] were the first to impose gradient weighting based on the local image structure with Werlberger et al. [20] combining the two in an anisotropic, image-driven Huber term. These methods make naive assumptions on the strength of each edge, given only its local structure and ignoring the global context of the scene. Such an approach could not suffice, as even rigidly moving objects can contain edges with arbitrary weights, as can be seen in Figure 2.

With the advent of deep learning, optical flow estimation methods using DNNs were proposed and the effort of the research community shifted to their optimization.

### 2.1. Implicit Regularization in Deep Neural Networks

The first work on optical flow estimation using CNNs was presented by Dosovitskiy et al. [21] who modeled it as a supervised learning problem. This model, FlowNet, was followed by a plethora of other works that sought to translate the powerful results of CNNs to optical flow. Subsequently, the proposed architectures were modified to better fit the nature of the problem, effectively providing an implicit regularization. The contributions included, among others, changes in learning rate scheduling, joint learning schemes, and pyramidal architectures.

Tran et al. [22] implemented a 3D convolutional architecture, while Ranjan et al. [23] built a light network that is effective on areas with large motion using a spatial pyramid scheme. This multi-scale approach eventually emerged as the most popular choice in many general backbone networks for feature extraction. Sun et al. [24] also utilized it to construct a differentiable cost-volume, which topped most benchmarks when combined with intermediate wrapping operations. Yin et al. [25] proposed a hierarchical probabilistic formulation, trying to estimate not only the flow vectors themselves, but also their local distribution. Such an approach is shown to be computationally intractable on a global basis and without a pyramid scheme.

FlowNet2 from Ilg et al. [16] focused on the merits of module stacking and curriculum learning. The authors claimed that progressively increasing the difficulty of training samples plays a critical role on accuracy; therefore, they proposed a stacked, progressively trained architecture with intermediate image warping operations, setting a new SoA. Hur et al. [26] claimed that stacked architectures benefit from reusing the same module for iterative residual refinement, instead of stacking independent modules. Hui et al. [27] tried to reduce the computational requirements of Ilg’s method. Their approach included warping operations on the feature pyramid level, along with a custom local convolution operation and cost volume construction. Sun et al. [28] investigated the impact of training procedure even further. By carefully scheduling the learning rate, they significantly improved the original FlowNet’s accuracy. Zhai et al. [29] made extensive use of dilated convolutions and residual blocks and demonstrated their detail-preserving properties.

### 2.2. Explicit Regularization in Deep Neural Networks

Explicit regularization is more commonly used in unsupervised learning methods, as the common reconstruction loss function is an unstable training objective. This can be implemented in various ways, such as a consistency loss term [30,31], utilizing flow predictions of classical methods for initialization [32], as well as an adversarial network [33]. The reconstruction error is naturally a bad means to achieve good results on occluded regions, as there is nothing to reconstruct. Liu et al. [34] enriched the training data with artificial occlusions, significantly improving occlusion performance. Of great interest in this area is the work of Yang et al. [35], where they proposed a Conditional Prior Network that regularizes the output based on the input. In essence, the network captures the possible motion space of a given single input image. Mun et al. [36] employs coupled spatiotemporal consistency checks in order to improve optical flow, depth, and ego motion.

Explicit regularization methods try to express some prior knowledge about the characteristics of real optical flow fields. This is typically implemented as an additive loss term. Unlike the works in [17,18,19,20], where only low level image features are used, our method utilizes high-level semantic object segmentation information to calculate the regularization term and in contrast to [33,35] its computation is simpler and requires no training.

### 2.3. Semantic Regularization

Several attempts have been made to exploit the interdependence of semantics and motion in order to apply some semantically-driven regularization. Ha et al. [37] propose a semantically guided loss that improves deformation estimation. Sevilla et al. [38] split the computation into three parts, based on the semantic category of each object. Their formulation considers three general kinds of moving entities, based on the irregularity of their motion: “planes”, “rigid” objects, and “stuff”. Such explicit approaches rely heavily on the granularity and accuracy of the segmentation [39]. Cheng et al. [11] tried to capture the interdependence between the tasks of semantic segmentation and optical flow estimation, jointly training a network for video object segmentation and optical flow, while employing late feature fusion. Wang et al. [40] used semantic masks for superpixel refinement and built a semantic-guided superpixel distance metric in order to improve sparse matching and flow estimation accuracy at object boundaries.

The correlation of optical flow and semantic masks has been explored in literature by jointly training a deep neural network to produce both of them. However, such approaches require significant structural modifications of the architecture and induce the additional burden of having to deal with multiple steps or training objectives.

## 3. Proposed Methodology

As already mentioned, regularization can be defined as any strategy employed to improve the training procedure of a neural network and to reduce its generalization error. In the relevant literature, different strategies have been followed, imposing restrictions and penalties that can lead to improved performance by either modifying the objective function or controlling the biases. Regularization techniques can pursue their aim among others by (1) encoding specific prior knowledge, (2) promoting a simpler solution, (3) transforming the problem into a determined one, or (4) combining multiple hypotheses to model the training data. In this work, we are mainly focusing on (1), exploring the use of semantically-driven local motion consistency in a semantic context, and (3), reducing the spatial invariance of the network to better determine the solution space. The exploration process performed for the proposed approaches is also presented, as it can offer significant insights in the discussion of the results.

### 3.1. Semantically-Driven Local Motion Consistency

Explicit regularization techniques impose some constraints directly on the output of the estimator, which in the case of deep neural networks, takes the form of a loss term. The simplest method is global consistency or global total variation, under which large, abrupt changes of the predicted motion field are penalized. Naturally, such a penalty decreases the accuracy in regions where the transition of the ground truth flow is sharp, such as on the boundary of two independently moving objects, as depicted in Figure 1.

**Figure 3:**
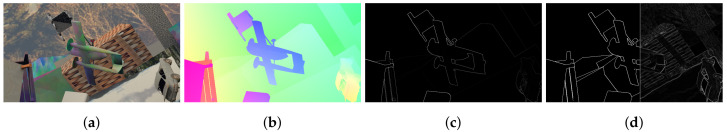
(**a**) Sample from FlyingThings3D dataset. (**b**) Ground truth optical flow. (**c**) Motion edges. Notice how the lion’s share of variation is concentrated on object boundaries. (**d**) Comparison of semantic edges with a typical intensity edge mask. Please note how much better the semantic edges correlate to the motion ones.

In such cases, a local regularization term that selectively regularizes motion consistent regions is required. These regions are bound by edges with sharp motion change, also known as motion edges. The identification of motion edges was performed by utilizing low level features such as intensity edges and gradients in their modeling [20], based on the observation that independently moving objects have distinct appearance and can be separated from an intensity edge. As it can be seen in Figure 3 though, such features are noisy predictors of motion edges, as arbitrary shape and texture exists within rigidly moving entities. Semantic segmentation, on the other hand, encompasses meaningful information about the rigidity and the actual boundaries of the objects. Figure 3 shows that edges from a semantic segmentation mask are strongly related to motion edges, and consequently we propose the use of such semantic edges as guides for our local, semantically-driven local smoothing. A more elaborate example can be found in Figure 2.

**Figure 4:**
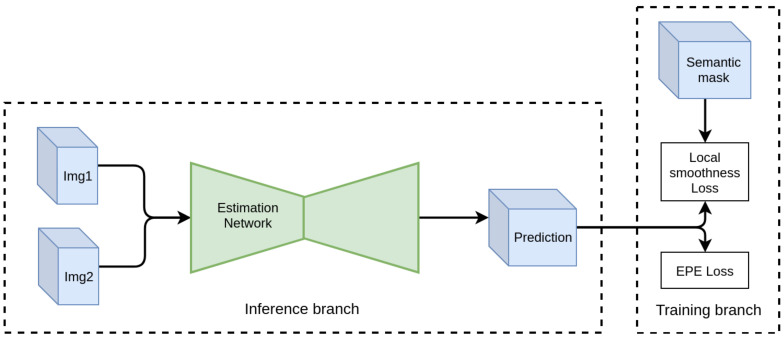
Illustration of the proposed training scheme. The additional loss component implicitly regularizes the predictions during training time, requiring no modifications at inference.

Given any optical flow estimation network, we modify the training branch by adding to the average endpoint error (AEE) loss the proposed Local Smoothing Loss, as depicted in Figure 4. No other modifications are performed to the architecture of the baseline network. Therefore, the inference procedure is not affected in terms of complexity or time. The added loss term penalizes motion inconsistency inside an object, guided by the semantic segmentation mask, while ignoring areas on the boundaries with other objects. The aim of this approach is to regularize the network, driving it to learn whether the image texture contains semantically important edges as opposed to simple intensity edges. Proper generalization is important as the network should make the discrimination without the help of a segmentation mask in the inference.

The loss is implemented as an element-wise multiplication of the consistency term with a binary mask that contains the edges extracted from the semantic instance segmentation. Motion spikes filtered by the mask are excluded from the consistency constraint, and are thus not regularized. Let us define the semantically-driven local motion consistency loss as

(1) $$l_{smooth}=\frac{\sum (\left(V_{x}+V_{y}\right)⊙M_{sem})}{\sum M_{sem}}$$

with $V_{k}$, where k∈{x,y}, being

(2) $$V_{k}\left(i,j\right)=\left|k_{i+1,j}-k_{i,j}\right|+\left|k_{i,j+1}-k_{i,j}\right|$$

*i* and *j* refer to the rows and columns of the optical flow field, $V_{x}$ and $V_{y}$ are the total variations for the two optical flow channels, $M_{sem}$ is the smooth patch boundary binary mask, and ⊙ indicates the Hadamard product. The total loss for our training procedure becomes

(3) $$l_{total}=l_{AEE}+\alpha *l_{smooth}$$

with alpha regulating the contribution of the semantically driven consistency term and $l_{AEE}$ the average endpoint error loss term. The endpoint error can be calculated as $∥V_{pred}-V_{gt}∥$ with $V_{pred}$ being the predicted optical flow and $V_{gt}$ the ground truth vector.

**Figure 5:**
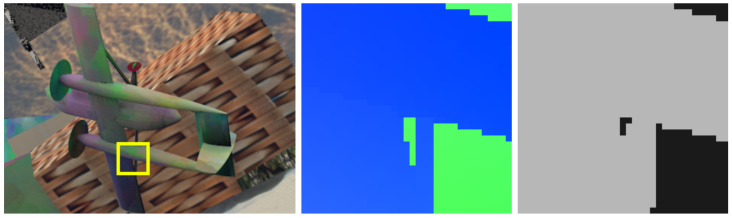
Sample from FlyingThings3D dataset with a cutout of its corresponding ground truth optical flow and object segmentation. Notice how the transition from the airplane to the background object happens at different positions in the two modalities.

$M_{sem}$ can be calculated by applying the derivative operator to an object instance segmentation mask, resulting in a binary mask that delineates object boundaries, as can be seen in Figure 2c. While ground truth object instance segmentation information is available for many flow datasets, some misalignment between the different modalities can exist, as it can be seen in Figure 5. In order to ensure good alignment between $M_{sem}$ and ground truth flow spikes, we apply a dilation operator after the derivation. A 3×3 dilation kernel proved sufficient, as almost all misalignment is up to two pixels.

### 3.2. Coordinates as Features

**Figure 6:**
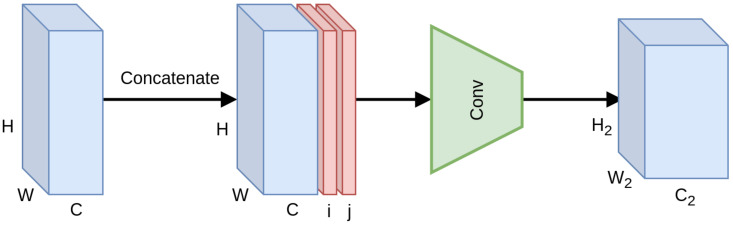
Schematic representation of the CoordConv module.

The idea of integrating pixel coordinates as features in CNN architectures was explored by Liu et al. [15]. They investigated the merit of utilizing pixel grid coordinates as input features at various stages of a CNN, introducing the CoordConv module (Figure 6). In this way, the output is being determined not solely by content-based feature values, but also from the position of those features on the grid, introducing spatial awareness to the convolution operation and breaking the celebrated translation invariance property. Furthermore, this information is propagated across layers, producing spatially-aware features across the whole network. In theory, the network can nullify the effect, learning to discard these features if necessary.

The methodology was tested on the toy problem of mapping coordinates in (x,y) Cartesian space to coordinates in an one-hot pixel space, significantly improving performance and convergence. While it seems intuitive to utilize coordinates as inputs on such a task, the expected effect is heavily domain-dependent and should be evaluated on a task by task basis. In image classification, for example, the impact of CoordConv is not significant, while in object detection, generative modeling and super-resolution [41], important improvements are noticed. Moreover, the selection of the convolutional layers to be changed is important for the performance of the network.

**Figure 7:**
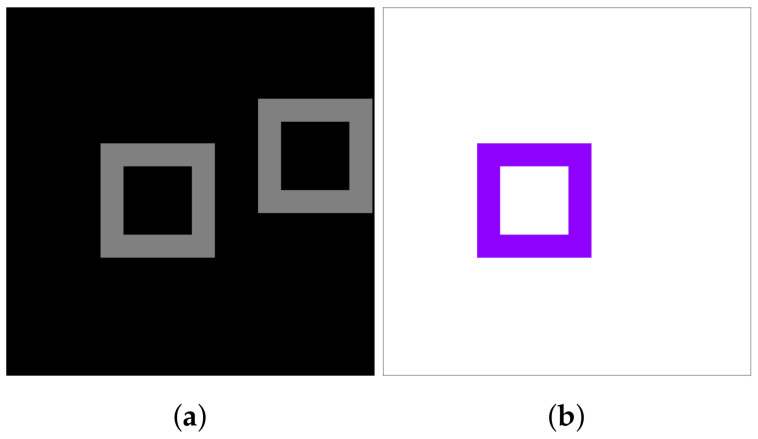
(**a**) Overlay of the images from sample of our toy dataset. (**b**) Ground truth optical flow.

We claim that the accurate pixel correspondence calculation required in optical flow estimation inherently relies on such a feature. We focus on proving our claim on both small and large scale experiments and architectures. We believe that the usage of pixel coordinates can be integrated in optical flow estimation in the future. To assess its fundamental merit, we test the impact of CoordConv on a toy optical flow estimation problem. We construct a simple dataset, containing 3136, 32×32 image pairs with 9×9 moving rectangles and their corresponding ground truth optical flow (Figure 7). Each rectangle can freely move within the image, while its distance from the center follows a uniform distribution.

**Figure 8:**
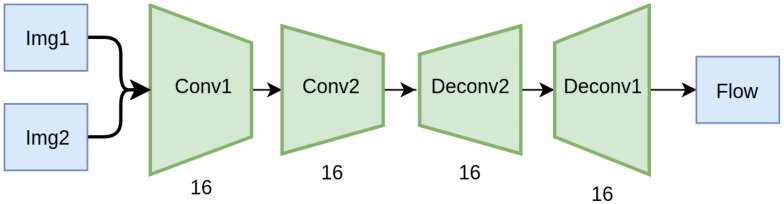
Overview of our baseline simple architecture. On the modified version CoordConv modules are appended to layers Conv1 and Deconv1.

**Figure 9:**
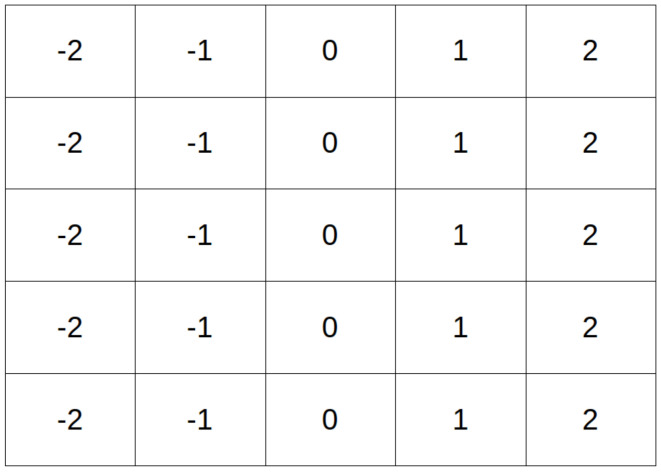
Example of an unnormalized x-coordinate tensor for a 5×5 pixels image.

We train a simple autoencoder-based network, depicted in Figure 8, on this simple dataset, with and without CoordConv layers, and compare their validation performance. The implementation of the CoordConv model is simple and straightforward. We concatenate a two-channel coordinate tensor at the input of the first and last layer. The coordinates are centered around zero and normalized in the range [−1, 1]. A trivial example of unnormalized coordinates for a 5×5 image is depicted in Figure 9.

**Figure 10:**
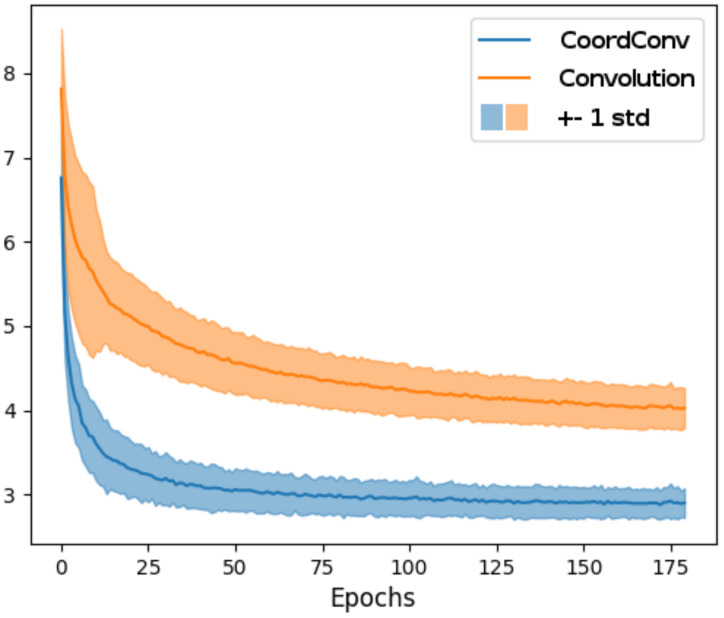
Magnitude and standard deviation of endpoint error for our simple networks trained on our toy dataset. Notice that the network with the CoordConv module converges significantly faster and on lower error values.

**Figure 11:**
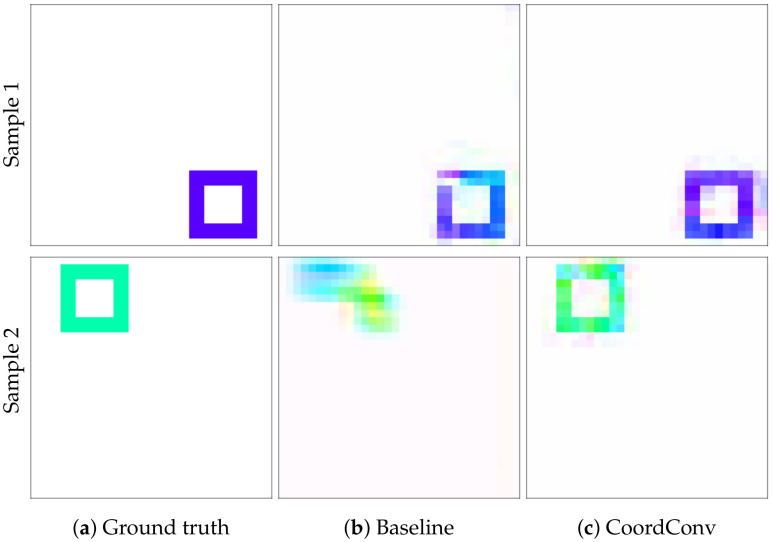
Validation samples after convergence on our toy dataset. Each row depicts a different sample and columns correspond to (**a**): ground truth optical flow, (**b**): baseline network prediction, and (**c**): CoordConv network prediction.

The training of the network using CoordConv converges after 85 epochs, in contrast to the baseline network which fails to converge even after 175 epochs (Figure 10). The experiment was repeated 100 times for validation. It is evident that training is more stable using CoordConv, with the majority of the experiments converging around the same error value, as indicated by the low standard deviation of the error curve. In Figure 11, the improvement is clearly depicted in the qualitative comparison of the example flows. Although the baseline network sometimes outputs fuzzy and inconsistent outlines, using CoordConv the output consistently follows the shape of the ground truth. This performance gap cannot be attributed to suboptimal hyperparameter values, with the relative difference between the two models remaining the same across a wide range of learning rates.

## 4. Experimental Evaluation

After demonstrating the theoretical merit of the proposed regularization techniques, in this section, they are extensively evaluated. FlowNet2 [16] has been selected as the reference architecture for the experimental evaluation, as its modularity allows for easier training, achieving high performance and accuracy. It comprises a stack of backbone modules, namely, FlowNet C and FlowNet S, which implement the well known encoder–decoder scheme. Together, they form a refining stack, as each module takes as input the previously computed flow and improves upon it. Overall, each added module takes as input the two images (Img1 and Img2), the previously estimated flow, the warped image Img2_warp_, and the error between Img2_warp_ and Img1. Img2_warp_ is computed using backward bilinear warping on Img2 using the flow field from the previous stage. As the inputs pass through the stacked modules of FlowNet2, the predicted flow gets progressively refined and approaches the ground truth, where Img2_warp_ should approximate Img1 and the error should reach zero.

**Figure 12:**
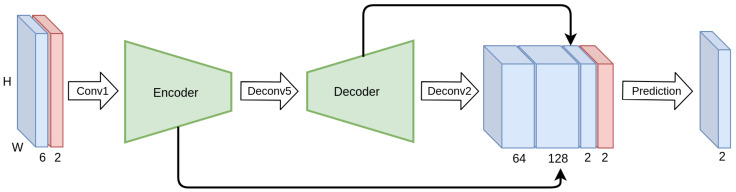
Overview of the modified FlowNet S architecture with added CoordConv modules. Grid coordinates (red) are concatenated before the first and last convolution.

**Figure 13:**
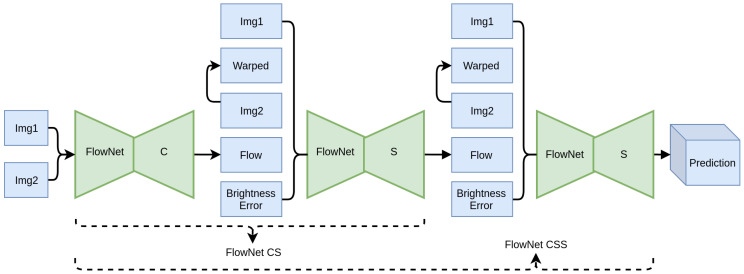
Overview of the network architecture. We utilize three stacked autoencoder refinement modules.

In order to evaluate our contributions, we apply the proposed regularization methods to FlowNet S and proceed with training the network, replicating the gradual training procedure of FlowNet2. The modified FlowNet S module is constructed as depicted in Figure 12. The evaluation is performed in two stages, as depicted in Figure 13. At the first stage, a stack comprising a FlowNet C module, followed by a FlowNet S is created, forming FlowNet2 CS. The contribution of the regularization based on (a) the semantically-driven motion consistency and (b) the coordinate feature layer, is studied comparing the baseline FlowNet S module and the proposed modified one. At the second stage, another FlowNet S module is added to the stack forming a FlowNet2 CSS and the proposed regularization methods are again assessed, both quantitatively and qualitatively, to prove the validity of our claims.

### 4.1. Implementation Details

The FlowNet2 building blocks were implemented in Pytorch, namely, FlowNet C and S. Due to the fact that we cannot reproduce accurately the augmentation process followed in the original work, we demonstrate results comparing our implementation and the modified version proposed. Both networks are trained identically, using the same train/test split ratio, training schedule (epochs), and learning rate.

The training of the models is performed using FlyingChairs [21], containing 22,872 samples, and FlyingThings3D [42], containing 80,604 samples. Although both of them are synthetic datasets that contain unrealistic scenes, they are widely used as training sets in optical flow estimation, as they contain a large number of samples, leading to good generalization. Throughout the training process we use the reference train–test splits, if provided, or assume random 80–20 train and test splits following standard practice.

Following the procedure proposed in the original implementation, each network is first trained on FlyingChairs (Long scheduling) and then on FlyingThings3D (Fine scheduling) for a total of 1.7 million iterations. Under the long schedule, the learning rate is halved every 200 k iterations after the 400 k mark and until reaching 1.2 M iterations. Fine scheduling covers the last 500 k iterations and the learning rate is, again, halved every 100 k iterations after the 200 k mark.

The evaluation is conducted on MPI Sintel [43], KITTI datasets [44,45], and Middlebury [46]. MPI Sintel is synthetic but contains plenty of dynamic scenes (1041 samples) with complex motion and photo-realistic effects. KITTI datasets are the sole available large-scale, realistic datasets, but they contain only 394 samples in total and the available ground truth is sparse. Finally, Middlebury consists of 4 synthetic and 4 natural samples with dense ground truth. The metrics used for evaluation are the widely adopted average flow vector endpoint error (AEE) and the outlier percentage (OP). For the latter, a pixel is considered to be an inlier if its endpoint error is <3 px or <5%.

We conducted our experiments on commodity hardware, i.e., a Linux-based PC with an NVIDIA 1080GTX GPU, 32 GBs of RAM, i7–6700 K CPU. For our implementation, we used Python 3.6 and the Pytorch library. The training hyperparameters are the same as in the baseline reference in order to ensure fair comparability of the results.

### 4.2. Exploratory Process

In this section, we explore the independent value of each of our contributions, by testing them on a smaller scale. We train FlowNet2 CS on FlyingChairs using different types of regularization.

Initially, the effectiveness of all consistency-based regularization schemes is evaluated. “No regularization” is considered as the baseline and it is compared against the use of global and semantically-driven local consistency as regularization constraints. The regularization factor *a* in (3) is set to 0.01 for all the experiments performed. This value leads to a relative weight of the regularization term to the main training objective of ≈1%. The value of *a* has a direct effect on the amount of smoothness of the produced optical flow. Small values of *a* induce results imperceptible to “no regularization”, whereas large values cause oversmoothing effects and hurt the accuracy of optical flow. We experimented with values on a logarithmic scale (a∈{0.001,0.01,0.1}) and found that a=0.01 achieves improvement in both training objectives. Due to processing and timing constraints, we did not perform extensive parameter grid search to find the optimal value (which can be dataset-specific). Different values of *a* in the same range are expected to have small performance deviations. The results in terms of AEE and OP are depicted in Table 1.

The results show that methods using regularization based on both the global and local consistency loss are surpassing the baseline on 2 of the 3 evaluation datasets. The proposed semantically driven local consistency loss leads to the best results on KITTI 2012 and 2015, both in terms of AEE and OP. On Sintel, both regularization methods are producing a lower AEE than the baseline but a higher Outlier Percentage (OP). This is attributed to the fact that Sintel has similar characteristics to the training set and small non-regularized networks tend to overfit to the training data. The use of the semantically driven local consistency loss produces results superior to the global one in all cases. As it is validated in the next sections, fine-tuning the network with a dataset rich in semantic entities generalizes better and the regularized network has better performance even in simple datasets. Similarly, networks with a bigger capacity perform better at all cases. Overall, regularizing the network using consistency constraints improves the optical flow estimation over the baseline.

The qualitative difference between the two methods, is highlighted in Figure 1. It is evident that the flow on the silhouette of the object is crispier with visibly less blur when using the semantically driven local consistency loss, in contrast to the oversmoothed boundaries of the globally regularized example. In complex scenes that involve many objects, the result is visibly better combining sharp edges and smooth flow within the objects.

After evaluating the effect of our novel consistency constraint as a regularizer, we move forward to incorporate the proposed modification of FlowNet S with the CoordConv layers. FlowNet2 CS and the proposed modified network are trained following the procedure described in Section 4.1, performing both the long and the fine scheduling training stages. The results for each stage are reported in Table 2.

The modified FlowNet2 CS architecture benefits from our contributions, outperforming the baseline both in terms of AEE and OP in all used datasets. The improvement is consistent in both training stages and is more evident in the realistic KITTI 2012 and 2015 datasets, where the proposed network improves the baseline by 0.5 to 1 pixel in terms of AEE and by 1 to 2.5 points in terms of OP. Moreover, comparing Table 1 and Table 2, the additive improvement of the two methods can be seen, validating their complementarity.

### 4.3. Full-Scale Experiments

In the previous section, the merit of both implicit and explicit regularization has been demonstrated on the FlowNet2 CS model, as well as their complementarity. It has not yet been explored, though, how the proposed regularization methods are performing on deeper architectures that include more stacked modules. It has been shown that increased depth acts as an inherent regularizer, and therefore it is important to analyze the behavior of the proposed methods on a deeper model. For this purpose, we employ the FlowNet2 CSS architecture that stacks another Flownet2 S module on top of FlowNet2 CS. The network is trained following the same procedure, employing both a long and a fine training schedule. The evaluation is performed on four datasets and the results are presented in Table 3 in comparison to the baseline FlowNet2 CSS.

The proposed modified FlowNet S module is attached to the CS stack forming FlowNet2 CSS. With the weights of first stage fixed, training is continued on FlowNet2 CSS, following the same procedure. Both a long and a fine training schedule are employed and the results are shown in Table 3.

Quantitatively, the proposed CSS network outperforms the baseline FlowNet2 CSS on all available datasets by a significant margin. KITTI sets are the most representative ones of a real world scenario and constitute the de facto test for modern algorithms, while Middlebury is a renowned dataset with real samples. It is worth noticing that the results of the proposed CSS are lacking in performance when trained only with the simple FlyingChairs dataset, qhereas training on FlyingThings3D drastically improves the results. Despite the fact that stacking is implicitly regularizing the network, the proposed approach is further improving the results increasing the lead from the baseline in the KITTI datasets.

**Figure 14:**
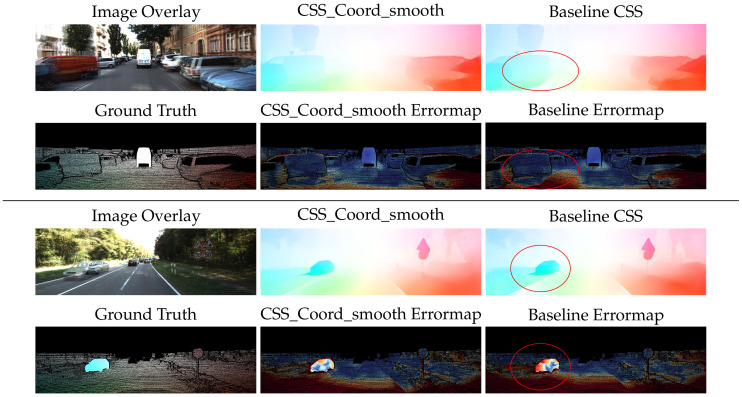
Qualitative results on the KITTI2015 dataset. The network with our contributions produces more accurate, detailed, and smooth results than that of the baseline network. Error magnitude increases transitioning from dark blue to yellow and red.

**Figure 15:**
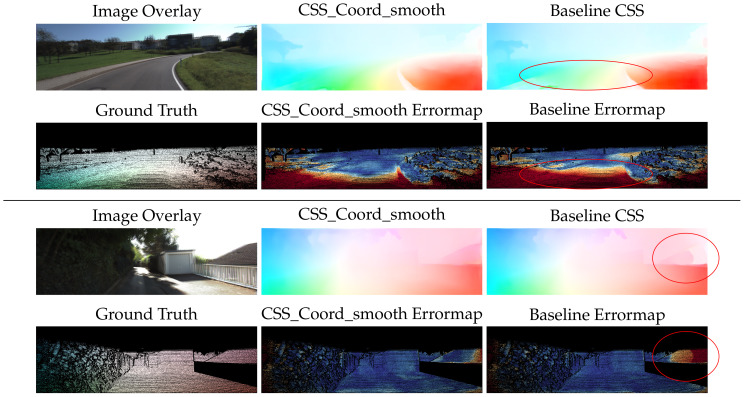
Qualitative results on the KITTI 2012 dataset. The network with our contributions produces more accurate, detailed, and smooth results than that of the baseline network.

**Figure 16:**
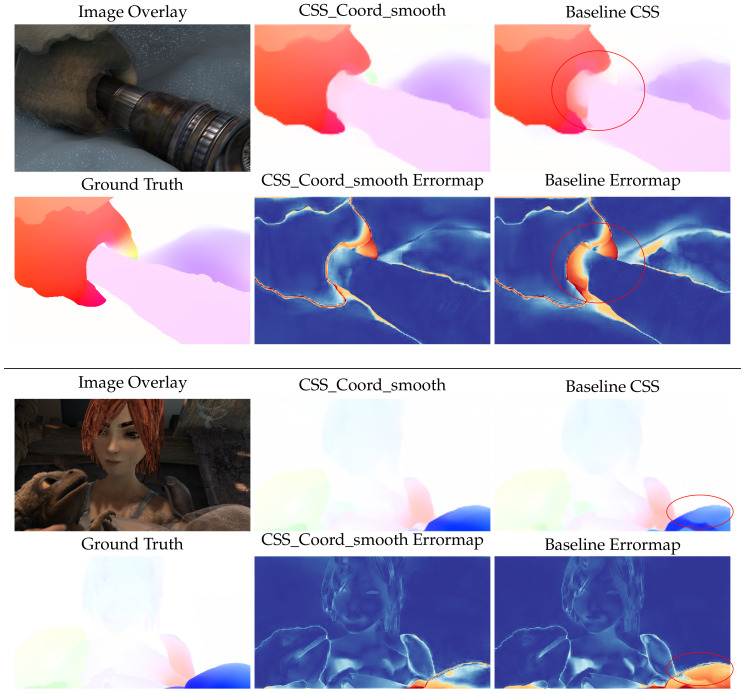
Qualitative results on MPI Sintel dataset. Although the baseline network overfits and presents lower error magnitudes on this synthetic dataset, our method maintains its qualitative edge, producing flow vectors that adhere to object boundaries.

**Figure 17:**
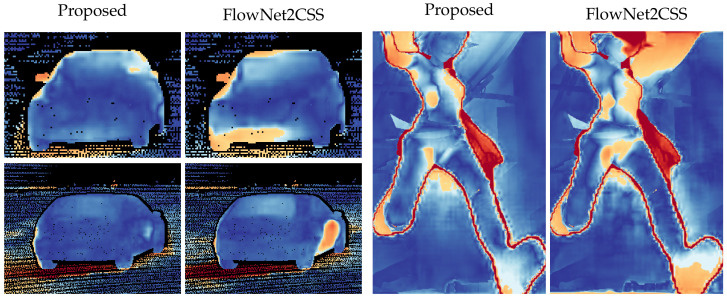
Closeup of differences at object boundaries between errormaps of the proposed method and FlowNet2CSS.

Qualitative inspection of the results depicted in Figure 14 and Figure 15 validates the numerical improvements seen in the results. It is clear that the proposed network handles the inherent motion consistency of planes (e.g., roads and pavements) and objects better than the baseline. In Figure 16, where scenes from Sintel are depicted, the results of the proposed network maintain better sharpness on object boundaries with significantly less flow leakage between objects in the highlighted regions. The objects are delineated in a more clear and consistent manner, as also demonstrated in Figure 17.

**Figure 18:**
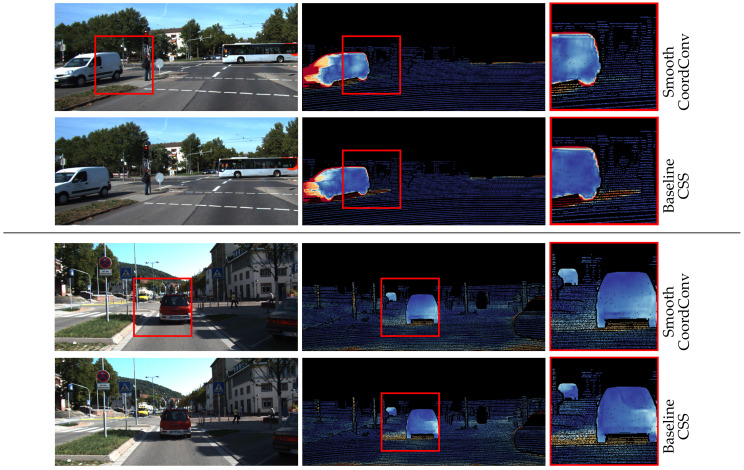
Typical examples of improved shadow handling with our approach. Shadows that appear to move with their casting objects are better mapped to the background.

Shadows constitute a particularly interesting case for optical flow algorithms, because they break the brightness constancy constraint. More specifically, unsupervised methods that are trained with reconstruction loss produce erroneous flows at shadows, as they appear to move together with the object casting them. This is another manifestation of the flow leakage problem, as the motion vectors of the moving object erroneously leak to its shadow. The proposed method enforces flow consistency within semantic entities, reducing leakage and errors in those areas. Examples of this behavior can be seen in Figure 18.

## 5. Discussion

The aim of this work was to explore means of regularization that can improve optical flow estimation networks in terms of generalization and ease of training. Towards this objective, two regularization strategies were proposed and evaluated.

The first one includes the infuse of semantic information in the training process to enable a better understanding of the scene context and, subsequently, to achieve better flow estimation at inference. The proposed semantically-driven local consistency regularization method utilizes ground truth semantic information to learn to implicitly identify the semantic edges of an object and better deduce the motion vectors around them. In the context of the optical flow estimation, semantic edges proved to be a better approximation of the real “motion edges” of an object compared to edges from a traditional edge detector, as used in the literature. The proposed method is highly versatile and can be easily integrated into existing training pipelines without modifications in the inference step.

During the experiments, it was noticed that the accuracy of the results was highly related to the quality of the semantic information. The available ground truth semantic segmentation is not fully aligned with the respective images, causing inconsistencies when combined. It is believed that training with finer segmentation masks would prove even more beneficial. Moreover, the use of instance segmentation masks could further improve the results.

The second method proposed deals with the use of pixel coordinates to regularize the training process. An implicit regularization method using the normalized coordinates of each motion vector as a feature was also proposed and experimentally assessed for optical flow estimation. This method is adding spatial awareness to the data input of the network, breaking the spatial invariance properties of the CNN. Extensive experimental evaluation proved that this is beneficial for the training process improving speed and reducing variance, while converging to a better solution. Although this regularization method requires some architectural modifications, minimal processing overhead is added during the inference stage.

Finally, it was proven that both proposed methods are complementary and can be combined to further improve the results. The well-established FlowNet2 was used as a reference architecture to evaluate the added value of the proposed solution. The accuracy of the baseline flow estimation network is improved on both synthetic and real datasets, while minimally affecting the underlying architecture.

## Figures and Tables

**Table 1:** Global vs. local smooth results trained on FlyingChairs with long scheduling.

	Sintel Clean		KITTI 2012		KITTI 2015	
Method	AEE	OP	AEE	OP	AEE	OP
FlowNet2 CS	2.89	**12.15**	8.07	41.22	16.05	48.53
Global Smooth CS	2.84	12.40	7.29	37.10	15.74	46.88
Local Smooth CS	**2.82**	12.37	**7.23**	**36.58**	**15.39**	**46.00**

**Table 2:** FlowNet2 CS results vs. baseline on different training steps.

	Sintel		KITTI 2012		KITTI 2015	
Method	AEE	OP	AEE	OP	AEE	OP
Long scheduling on FlyingChairs						
FlowNet2 CS	2.89	12.15	8.07	41.22	16.05	48.53
Proposed CS	**2.80**	**10.31**	**6.76**	**31.10**	**15.08**	**44.06**
Fine scheduling on FlyingThings3D						
FlowNet2 CS	2.27	8.42	4.96	21.43	12.11	34.22
Proposed CS	**2.22**	**8.12**	**4.42**	**20.20**	**11.15**	**33.00**

**Table 3:** FlowNet2 CSS results vs. baseline after the final training step.

Dataset		FlowNet2 CSS	Proposed CSS
Middlebury	AEE	0.55	**0.41**
	OP	2.15	**2.07**
Sintel clean	AEE	2.12	**2.02**
	OP	7.61	**7.02**
KITTI 2012	AEE	4.69	**4.01**
	OP	19.50	**17.76**
KITTI 2015	AEE	11.89	**10.66**
	OP	31.87	**30.52**
